# Accuracy in the Determination of Chlorinated Dibenzo-p-Dioxins and Dibenzofurans in Environmental Samples

**DOI:** 10.6028/jres.093.030

**Published:** 1988-06-01

**Authors:** L. L. Lamparski, T. J. Nestrick

**Affiliations:** The Dow Chemical Company, Michigan Applied Science and Technology, Laboratories, 574 Building, Midland, MI 48667

The analytical chemist involved in industrial trace analysis is frequently confronted with many varied problems. Programs to produce acceptable data can be divided into two classes depending upon the level of quality assurance that is required. Frequently, unvalidated analytical procedures can be used to generate data for screening programs or process control. On the other hand, validated methods with rigorous quality assurance guidelines are absolutely necessary for work involving product specifications, industrial hygiene, or regulatory matters.

When the analyst is asked to develop a trace analytical method, he must determine the end-use of the data. There are many parameters which must be factored into the analytical method. The analyst would like to build into the method the highest achievable sensitivity, accuracy, and reliability; and the customer wants the lowest cost and fastest analysis time possible. We can see then, from a practical viewpoint, the method development begins with a series of compromises. When implemented properly, these compromises can improve the overall quality of the method.

The American Chemical Society has published “Principles of Environmental Analysis” [Anal. Chem. 55, 2210 (1983)] to aid in designing analytical measurements on environmental samples. The relationship between the number of samples necessary to obtain data within a defined acceptable error, and the standard deviation of the method, is shown by the equation:

$$N=\frac{{\left(Z\sigma \right)}^{2}}{E},$$

where *N* equals the number of measurements necessary, *Z* equals the standard normal variate based upon the level of confidence, *σ* equals the standard deviation of the method, and *E* equals the tolerable error in the estimate of the mean. Obviously, for constant values of *Z* and *E*, *N* is proportional to *σ*^2^. Any changes in an analysis method that increase *σ* will result in an exponentially larger increase in *N.* When a high degree of accuracy is required, an analytical method with a small *σ* will have a smaller value of *N* and require fewer measurements. In fact, by building the highest possible degree of reliability into a method, it may be possible to actually decrease the amount of time necessary to run *N* analyses. The method reliability can be improved by eliminating or at least recognizing factors which can lead to either high or low results.

High results can be caused by contaminated internal standards or reagents, interferences in the sample, or matrix effects caused by co-extractives in the sample extract. We have taken precautions to minimize the potential for these problems in our method for the determination of chlorinated dibenzo-p-dioxins (CDDs) and dibenzofurans (CDFs) by addressing each area.

1. Isotopically-labeled internal standards can be contaminated with native analyte or with other labeled impurities which could potentially lead to high results. Prior to the use of any internal standard in a sample analysis, we perform an extensive series of GC-MS analyses to verify chemical identity, isotopic purity, chemical purity, and absence of analyte contamination. To date, ~40% of purchased internal standards have failed to meet the criteria for use in our method.
2. Positive reagent blanks can originate from poor equipment cleaning, environmental contamination of sample extracts, or from contamination of chemicals or adsorbents used in the sample preparation. We have imposed a rigorous quality assurance program which involves analysis of a reagent blank with every set of three samples. A positive reagent blank can result in rejection of data and reanalysis of samples after the source of contamination is identified and eliminated.
3. Interferences in sample extracts are minimized by application of identification criteria which include multiple liquid chromatographic separations and gas chromatography-mass spectrometry separation and measurement.
4. Matrix effects can cause high results by changing the mass spectrometer response. The use of an analyte specific clean-up is useful in minimizing this problem. Identification of the species producing the effect and application of specific clean-up steps can eliminate problems.

Low results can result from incomplete analyte extraction, loss or degradation of the analyte during sample clean-up, or matrix effects. These problems can be eliminated by a careful evaluation of each of the steps employed in the sample preparation.

1. Extraction efficiencies can be improved by dissolving the sample matrix whenever possible and determining the optimum solvent for analyte extraction. When dissolution of the matrix is not practical, an exhaustive extraction procedure with an optimum solvent should be used.
2. Loss or degradation of the analyte can be controlled by determining the recoveries of all analytes through each clean-up step. An understanding of the chemical and physical properties of the analyte will not only improve recoveries, but also can allow optimization of each clean-up step.
3. Matrix effects leading to low results generally are caused by exceeding the capacity of the clean-up procedure for either analytes, related compounds, or coextractive species. This “overloading” can change the chromatographic retention of the analyte. In general, these effects can be minimized by decreasing the sample size or using a high-capacity pretreatment step to remove the coextractives.

Application of these precautions can result in a method which does not generate false positive or false negative results. A recent collaborative study to determine fortified levels of CDDs and CDFs in human adipose tissue at 5–50 pg/g has been completed by eight laboratories highly experienced in the determination of CDDs and CDFs [Albro et al., Anal. Chem. **57**, 2717 (1985)]. By implementing the practices described above, laboratory 2 avoided generating either unaccountably high or low results (table 1).

The apparent drawback of the laboratory 2 method is the relatively long analysis time per sample. However, when the standard deviation of the recoveries for each laboratory is calculated and used in the equation to determine the relative time to analyze *N* samples (measurements necessary to yield data of defined statistical reliability), method 2 can actually generate data with a specified precision in the shortest time.

## Figures and Tables

**Table 1:** Interpretation of recovery data from CDD/CDF collaborative study

	Lab 1	Lab 2	Lab 3	Lab 4	Lab 5	Lab 6	Lab 7	Lab 8
Avg. analysis time days/sample	0.9	2.6	0.6	1.0	1.0	1.5	–	2.5
Number of values unaccountably high	4	0	2	6	2	3	16	3
Number of values unaccountably low	0	0	0	1	10	8	7	0

Avg. recovery	115%	97.7%	114%	147%	97.0%	50.3%	135%	151%
*σ* (S.D. rec.)	51%	19%	48%	94%	143%	39%	123%	64%
*n*	26	23	23	26	23	26	23	25

Rel. time to *N*	2.5	1	1.5	9.2	22	2.4	–	11

*N* ∝ *σ*^2^.

Relative time to $N=\frac{{\left(\sigma_{\text{Lab}}\right)}^{2}\times \text{days}/\text{sample}}{{\left(\sigma_{\text{Lab}2}\right)}^{2}\times 2.6\;\text{days}/\text{sample}}$.

